# Signal Transduction Pathways in the Pentameric Ligand-Gated Ion Channels

**DOI:** 10.1371/journal.pone.0064326

**Published:** 2013-05-08

**Authors:** David Mowrey, Qiang Chen, Yuhe Liang, Jie Liang, Yan Xu, Pei Tang

**Affiliations:** 1 Department of Anesthesiology, University of Pittsburgh School of Medicine, Pittsburgh, Pennsylvania, United States of America; 2 Department of Chemical Biology and Pharmacology, University of Pittsburgh School of Medicine, Pittsburgh, Pennsylvania, United States of America; 3 Department of Structural Biology, University of Pittsburgh School of Medicine, Pittsburgh, Pennsylvania, United States of America; 4 Department of Computational and Systems Biology, University of Pittsburgh School of Medicine, Pittsburgh, Pennsylvania, United States of America; 5 Department of Bioengineering, University of Illinois at Chicago, Chicago, Illinois, United States of America; Yale School of Medicine, United States of America

## Abstract

The mechanisms of allosteric action within pentameric ligand-gated ion channels (pLGICs) remain to be determined. Using crystallography, site-directed mutagenesis, and two-electrode voltage clamp measurements, we identified two functionally relevant sites in the extracellular (EC) domain of the bacterial pLGIC from *Gloeobacter violaceus* (GLIC). One site is at the C-loop region, where the NQN mutation (D91N, E177Q, and D178N) eliminated inter-subunit salt bridges in the open-channel GLIC structure and thereby shifted the channel activation to a higher agonist concentration. The other site is below the C-loop, where binding of the anesthetic ketamine inhibited GLIC currents in a concentration dependent manner. To understand how a perturbation signal in the EC domain, either resulting from the NQN mutation or ketamine binding, is transduced to the channel gate, we have used the Perturbation-based Markovian Transmission (PMT) model to determine dynamic responses of the GLIC channel and signaling pathways upon initial perturbations in the EC domain of GLIC. Despite the existence of many possible routes for the initial perturbation signal to reach the channel gate, the PMT model in combination with Yen's algorithm revealed that perturbation signals with the highest probability flow travel either via the β1–β2 loop or through pre-TM1. The β1–β2 loop occurs in either intra- or inter-subunit pathways, while pre-TM1 occurs exclusively in inter-subunit pathways. Residues involved in both types of pathways are well supported by previous experimental data on nAChR. The direct coupling between pre-TM1 and TM2 of the adjacent subunit adds new insight into the allosteric signaling mechanism in pLGICs.

## Introduction

Vertebrate pentameric ligand-gated ion channels (pLGICs) regulate ionic conductance in nerve cells and play an important role in fast synaptic signal transduction [1], [2]. They are formed by five homologous or identical subunits assembled around the central channel axis. Each subunit is composed of three structurally and functionally distinctive domains: an extracellular (EC) ligand-binding domain, a pore-forming transmembrane (TM) domain, and an intracellular (IC) domain that controls channel localization in the nerve cell and modulation effects of second messengers, but may not be essential for channel assembly and function [3]. Agonist binding to the orthosteric site in the EC domain of pLGICs allosterically triggers conformational changes and allosterically activates the channels so that ions can pass through the cell membrane. How the signal of agonist-binding in the EC domain is propagated to a remote channel region has been studied extensively on nicotinic acetylcholine receptors (nAChRs) in the past [4]–[7]. It remains an open subject for investigation as to whether there are common activation or deactivation signal pathways shared by all pLGICs.

The bacterial pLGIC from *Gloeobacter violaceus* (GLIC) is a cationic homo-pLGIC [8]. The crystal structures of GLIC [9], [10] show a common scaffold with the vertebrate pLGICs, such as nicotinic acetylcholine receptors (nAChRs) [11], except without an IC domain. Opening of the GLIC channel is triggered by extracellular protons [8], but it is unclear which titratable residues are responsible for the GLIC activation. Similar to nAChRs [12], GLIC can be reversibly inhibited by general anesthetics in a concentration dependent manner [13]–[15]. Recent X-ray crystallographic studies revealed anesthetic binding sites not only in the upper part of the TM domain within each subunit [14], but also at the interface of two adjacent subunits in the EC domain [13]. The high resolution structures and well defined anesthetic binding sites provide the opportunity to critically examine how perturbations on titratable residues of GLIC modulate the functional status of the channel and how anesthetic binding allosterically inhibits GLIC currents without blocking the channel.

Introducing a Markovian process into coarse-grained models has offered opportunities to assess signal propagation in proteins [16]–[22]. The perturbation-based Markovian transmission (PMT) model [21] is particularly effective for probing how different parts of a macromolecular machine respond to signal perturbation that is either due to ligand binding or site-specific mutations. It characterizes the dynamic response of all residues in the protein over the time course from the initial perturbation to equilibrium. It can identify key signal-mediating residues that can be readily validated experimentally [4]–[7].

In this study, we investigated signal transmission in GLIC from the EC domain to the TM domain of GLIC upon two different stimuli. The first one is at the C loop region, where residues E177, D178, and R179 potentially form salt bridges with residues K148 and D91 at the complementary site of an adjacent subunit. We performed mutations (D91N, E177Q, and D178N, termed the NQN mutation) to remove the potential of salt bridges. Perturbation to GLIC due to the NQN mutation was evidenced in our crystal structure and functional measurements as presented below. The second perturbation site at the EC domain is below the C loop, where the anesthetic ketamine was found to bind to an existing inter-subunit pocket and inhibit GLIC current in a concentration dependent manner [13]. While the functional relevance of these perturbation sites is proven, it needs to be further clarified how the perturbation signal propagates from the EC domain to the channel gate. Here we used the PMT model to identify crucial signaling paths within a subunit and between adjacent subunits of GLIC. The resulting information will facilitate our understanding of the mechanisms of allosteric action in pLGICs.

## Results and Discussion

### Two functionally relevant sites at the EC domain of GLIC

**Figure 1:**
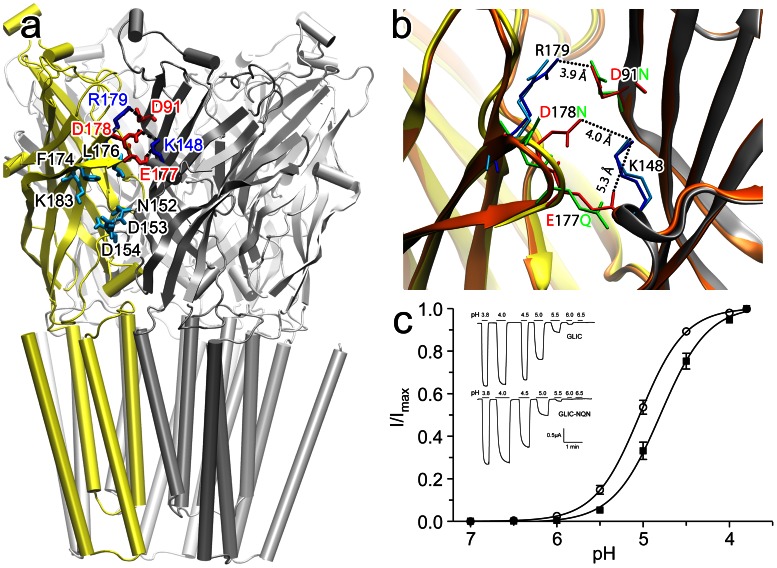
Functionally relevant sites in the EC domain of GLIC. (**a**) Residues for the NQN mutation (D91N; E177Q; D178N) and the complementary basic residues (R179 and K148) for salt bridge formation are highlighted in red and blue, respectively. Residues involved in the ketamine binding site (F174, L176, K183; N152, D153, D154) are highlighted in cyan. (**b**) The C loop region of the crystal structure of the NQN mutant (orange; PDB code: 4IRE), showing an outward movement of the C loop in comparison with the wild type GLIC (yellow and gray; PDB code 4F8H) due to removal of salt bridges in the mutant. R179 and K148 are shown in blue and cyan sticks for GLIC and the NQN mutant respectively. D91N, E177Q, and D178N are shown in red and green sticks, before and after the mutation, respectively. The salt bridge distances in GLIC are highlighted. Note the enlarged gap after the mutation. No hydrogen bonds could be formed for the mutated residues. (**c**) Two-electrode voltage clamp measurements on *Xenopus laevis* oocytes expressing the NQN mutant (solid square) and the wild type GLIC (open circle). The half maximal effective concentrations (EC_50_) for the mutant and GLIC are pH 4.80±0.03 (n = 13) and 5.04±0.02 (n = 10), respectively. The EC_50_ difference between the wild type GLIC and the NQN mutant is statistically significant (p<0.0001). Error bars represent standard error from the mean. The inserts are the representative traces for GLIC and the NQN mutant.

Two functionally relevant sites at the EC domain of GLIC (Fig. 1a) were chosen for investigating how perturbation signals are transmitted from the EC domain to the channel gate.

One site is at the C loop region, where the inter-subunit salt bridges (E177-K148, D178-K148, and R179-D91) are observed in the crystal structures of the open-channel GLIC [13], [23]. In order to understand the functional role of these salt bridges, we performed the NQN mutation (D91N, E177Q, D178N) to eliminate the salt bridges, crystallized the NQN mutant, and solved its structure (PDB: 4IRE) to a resolution of 3.19 Å (Table 1). The overall structures of the NQN mutant and GLIC are nearly the same (RMSD ∼0.5 Å) and show an open channel conformation. However, the C loop of the NQN mutant shows an outward movement and the interfacial gap in the C loop region, measured by side chain displacement of D178N, widens 3 Å (Fig. 1b). The NQN mutation removed the salt bridges but did not generate hydrogen bonds. To compare the conformational stability before and after the mutation, we calculated free energies for the inter-subunit interface in the crystal structures of the wild type GLIC and the NQN mutant. The resultant free energies of –29 kcal/mol and –26 kcal/mol for GLIC and the NQN mutant, respectively, suggest that removing the salt bridges at the subunit interface destabilized the open channel conformation. Functional measurements of the wild type GLIC and the NQN mutant provide results consistent with the free energy calculations. The mutation shifted the EC_50_ from pH 5.0 in the wild type GLIC to pH 4.8 in the NQN mutant (Fig. 1c). Statistical analyses confirmed that the EC_50_ difference between the wild type GLIC and the mutant was significant with p<0.0001. Apparently, more protons are required for channel activation to compensate for destabilization of the open-channel conformation due to the absence of the inter-subunit salt bridges.

**Table 1:** Data collection and refinement statistics.

Data collection and process	
Beamline	SSRL BL12-2
Wavelength (Å)	0.9795
Space group	C2
Unit cell (Å)	182.0, 133.6, 161.4
β (°)	102.6
Resolution (Å)	29.86–3.19 (3.36–3.19)
R_merge_(%)^a^	6.8 (70.7)
Completeness (%)^a^	97.5 (92.9)
<I/σ>^a^	14.0 (1.8)
Unique reflections^a^	61417 (9335)
Redundancy^a^	3.8 (3.7)

Refinement statistics	
Resolution (Å)	29.86–3.19
No. Reflections (test set)	61291 (999)
R_work_/R_free_	0.204/0.243
Non-H protein (ligand) atoms	12686 (754)
<B-factors> (Å^2^)	
Protein	87.3
Detergents	107.5
Lipids	121.0
Solvent	93.5
R.M.S. Deviations	
Bond lengths (Å)	0.009
Bond angles (degrees)	1.3
Rotamer outliers (%)	5.3
Ramachandran outliers (%)	0.19
Ramachandran favored (%)	97.17
PDB code	4IRE

a Values in the parentheses are for highest-resolution shell.

The other relevant location is the ketamine-binding site [13], which we identified previously in a 2.99-Å resolution X-ray structure of the GLIC-ketamine complex (PDB:4F8H). Ketamine binds to an inter-subunit cavity, which is lined by residues F174, L176 and K183 on the principal side and N152, D153 and D154 on the complementary side. The ketamine binding site is partially overlapped with the homologous antagonist-binding site in pLGICs. The functional relevance of the ketamine site was determined by profound changes in GLIC activation upon cysteine substitution of the cavity-lining residue N152. The functional relevance was also evidenced by changes in ketamine inhibition upon the subsequent chemical labeling to N152C.

These structural and functional data highlight functional relevance of the two sites and provide the experimental basis for initial perturbation in PMT calculations as presented below.

### Time-dependent transmission of perturbation initiated at the NQN mutation site and the ketamine-binding site

**Figure 2:**
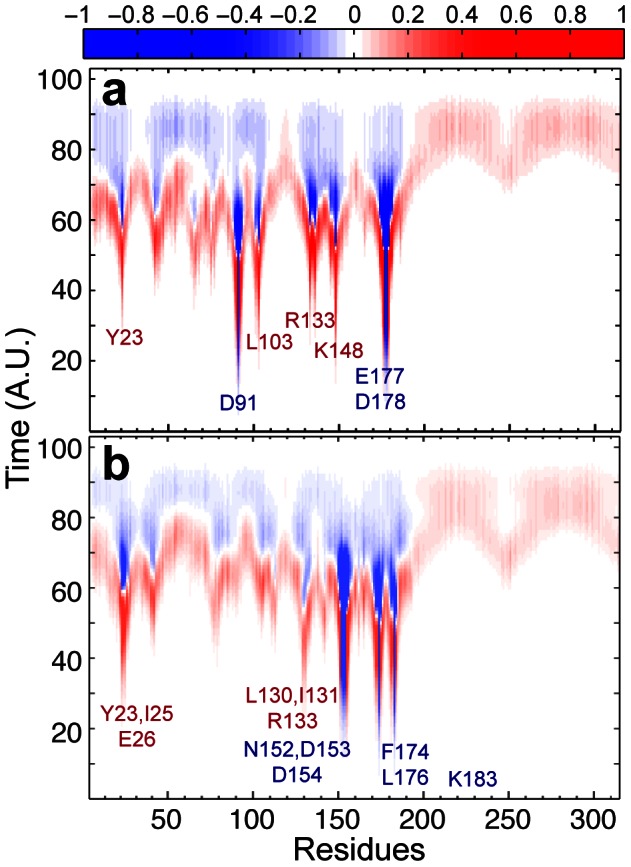
Trajectories of the probability flux over time for each residue upon different initial perturbations. (**a**) Initial perturbation at the NQN mutation site; (**b**) initial perturbation at the ketamine-binding site. The color denotes the normalized intensity of the probability flux (Eq. 1 in the method section). The positive and negative signs describe the net signal flow into and out of the residue, respectively. The time axis is in arbitrary unit. The initially perturbed and immediately affected residues are labeled in blue and red, respectively.

To reveal the allosteric signaling pathway in GLIC, we placed an initial perturbation of uniform strength on residues shown in Fig. 1a for the NQN mutation or ketamine binding within the PMT model. The time-dependent probability flux, defined in Eq. 1, was calculated for each selected scenario of initial perturbation site (Fig. 2). The pertubation originated from the NQN mutation site was transmitted immediately to Y23, L103, R133, and K148. Among them, R133 and K148 form intra- and inter-subunit salt bridges with D178 and E177, respectively. The perturbation at the ketamine binding site was transmitted rapidly to a cluster of residues in β1 (Y23, I25, E26) and β6 (L130, I131, R133). These residues are mostly in close contact with the perturbed sites. As time proceeds, more and more residues in the EC domain experienced the positive probability flux (colored red in Fig. 2). The positive probability flux occured in the TM domain when most residues in the EC domain experienced the negative probability flux (signal moved away, colored blue in Fig. 2).

**Figure 3:**
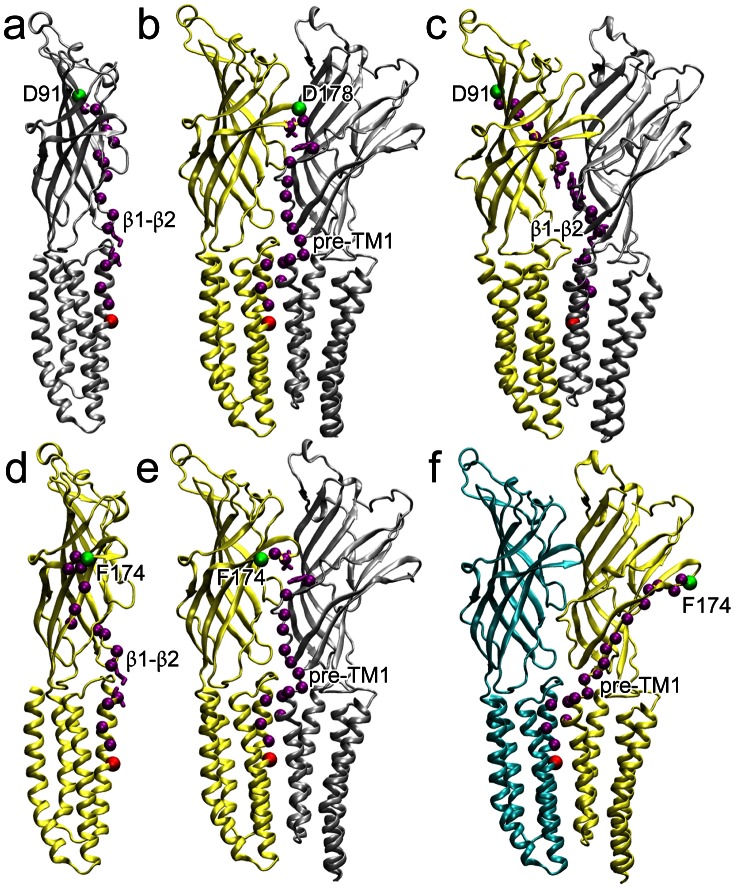
Paths with the highest probability to reach the channel gate (I233; 9′) under different initial perturbations in GLIC. (**a**) The path within a subunit upon perturbation to D91 of the NQN mutation; (**b**) the path between D178 of the NQN mutation site and I233 (9′) of the same subunit showing an inter-subunit pathway; (**c**) the path between D91 of subunit B and I233 (9′) of subunit A; the perturbation to F174 of the ketamine binding site shows both (**d**) intra- and (**e**) inter-subunit paths for signal starting and ending in subunit B; (**f**) the path between F174 of subunit B and I233 (9′) of subunit C. The perturbation starting and ending points are shown in green and red spheres, respectively. The pathways are highlighted in purple spheres. Subunits A, B, and C are colored silver, yellow, and cyan, respectively. All calculations were performed using Yen's algorithm.

The two initial perturbation sites share similar overall patterns of the probability flux in the TM domain. The signals reached pre-TM1, the TM2-TM3 linker, and the C-terminus of TM4 before they propagated to other parts of the TM domain. The residues immediately affected by the perturbation in the EC domain were clearly identified, but specific signaling paths became obscured as the signal diffused through the protein. To trace the paths between the initially perturbed residues and the channel gate residue I233 (also named 9′, a commonly presumed hydrophobic gate residue), we used Yen's algorithm [24] that outputs the most likely paths based on the probabilities stored in the Markovian transmission matrix. The pore-lining residues other than 9′ were also tested as target residues and produced the same paths as observed for the target 9′. There were a total of three and six initially perturbed residues for the NQN mutation site and ketamine-binding site, respectively. For each of the perturbed residues involved in the NQN mutation site (D91, E177, D178) and ketamine binding site (N152, D153, D154, F174, L176, K183), 10 signal paths with the highest probability were determined using Yen's algorithm [24]. The signal starts at the perturbed residue and ends at the channel gate residue I233. For completeness, three scenarios following each perturbation were considered, assume all signals start in subunit B: (1) signal starts and ends within subunit B; (2) signal starts in subunit B and ends in subunit A; (3) Signal starts in subunit B and ends in subunit C. In total, 270 paths were obtained (9 initial perturbations, 10 paths of highest probability for each perturbation, 3 different scenarios for the ending point). Many of the observed signal pathways are degenerate. However, the emerged pathways of the highest probability for signal transduction from the EC domain to the channel pore in our analysis (Table S1) reveal the involvement of two critical regions. The first one is the β1–β2 loop (also named loop 2) that couples with the C-terminus of TM2 (Figs. 3a and 3d). The second one is pre-TM1 that often mediates signaling between subunits (Figs. 3b, 3e, and 3f).

### The paths via the β1–β2 loop

Paths involving the β1–β2 loop can be either within a subunit or between adjacent subunits. For the intra-subunit signaling path, the perturbation signals resulting from the NQN mutation and ketamine binding initially travel via different routes, but eventually emerge at the β1–β2 loop, and further propagate along the same path to the channel gate. For example, as shown in Figs. 3a and 3d, the initial perturbations at D91 and F174 have two respective paths at the beginning: (i) D91, V90, V89, D88, A87, D86, S107, A108, R109**→** T36 of the β1–β2 loop; and (ii) F174, N173, A172, K183, Y129, H127, L126**→** F37 of the β1–β2 loop. Once the signal reached the β1–β2 loop, the rest of the path follows: F37, T36, A34, K33, T244, E243, N239, I236, I233(9′).

Assuming perturbations start in subunit B, inter-subunit paths involving the β1–β2 loop are observed for signals ending in either subunit A or subunit C. We note variations in the signal path when the initially perturbed residue or the ending subunit are varied (Table S1), but the involvement of the β1–β2 loop was observed in 66% of 270 paths identified by Yen's algorithm (Table S2). More details are provided in the supporting materials.

The important role of the β1–β2 loop in the channel function has been well documented by experimental studies. Mutagenesis in the mouse α1 subunit of nAChR and subsequent single channel electrophysiology measurements in the nAChR by Auerbach's group showed that residues in the β1–β2 loop, homologous to GLIC D32 (α1-E45) and K33 (α1-V46), are critical for channel gating [25], [26]. Sine's group also found the critical role of α1-E45 and α1-V46 in the channel gating of the human nAChR [6]. Furthermore, residues at the C-terminus of TM2 of the mouse nAChR, homologous to GLIC E243 (α1-V261) and T244 (α1-E262), were found in the same gating block (Φ ∼ 0.8) as the residues in the β1–β2 loop [27]. They are significantly coupled to channel gating [27]. More comparisons between our model predictions and experimental data on nAChR are provided in a specific section below.

### The paths via pre-TM1

The paths involving pre-TM1 were not observed as frequently as those involving the β1–β2 loop, but the significant occurrences of these paths (34% of the paths identified) make them worth noting. Unlike the β1–β2 loop that occurs in the signaling pathways both within a subunit and between adjacent subunits, pre-TM1 occurs exclusively in pathways across adjacent subunits. Assuming all perturbations start in subunit B, there are at least two types of paths involving pre-TM1. First, the initial perturbation signal (such as L176) traveled across subunit B, passed the pre-TM1 in the adjacent subunit A (Y23, N152, V155, F156, T158, G159, **Q193, Y194, F195, S196**, N200), and then propagated to the channel gate in subunit B (E243, N239, I236, **I233**), such as shown in Figs. 3b and 3e. Second, the initial perturbation signal traveled through pre-TM1 of subunit B (F174, N173, A172, P171, K170, V168, A167, T166, F165, S164, E163, I162, D161, **Q193, Y194, F195, S196**, N200) before reaching TM2 and the channel gate of subunit C (E243, N239, I236, I233), such as shown in Fig. 3f. Additional high probability paths involving pre-TM1 between a perturbed residue and the channel gate are provided in Table S1.

The involvement of pre-TM1 in signaling paths between the EC domain and the channel gate is not unexpected. Pre-TM1 covalently links the EC and TM domains. The functional contribution of pre-TM1 has been recognized in the past. However, the contribution was often attributed to the coupling with other loops at the EC-TM interface [6], [26], [28]–[30]. Mutagenesis, single-channel kinetic analyses, and thermodynamic mutant cycle analyses on the nAChR revealed energetic coupling among residues from pre-TM1, the Cys-loop, and the TM2–TM3 linker [28]. Specific interactions between pre-TM1 and the β1–β2 loop are shown in crystal structures of the mouse *α*1 nAChR extracellular domain [31] and GLIC [10], [23]. The functional coupling of pre-TM1 with the loop β1–β2 has been demonstrated in several experimental studies [26], [28], [30]. It was proposed that the coupling of pre-TM1 to the TM2–TM3 linker constitutes a principal transduction pathway [6], [29]. Our analysis here reveals a novel coupling mode of pre-TM1, in which pre-TM1, in conjunction with the C-terminal end of TM1, can directly transduce signals to TM2 and the channel gate of the adjacent subunit. This newly identified coupling is more direct and probably more effective for pre-TM1 to convey signals from the EC domain to the channel gate. In addition, since the coupling is between adjacent subunits, it facilitates communications and cooperative action among subunits.

It is worth noting that among all four TM helices, the TM2 conformation is the most sensitively correlated to the channel state as indicated in the crystal structures [9], [32] and in MD simulations [33]. The TM1 conformation is the second most sensitive to the channel state [9], [32], [33]. The direct coupling of pre-TM1 N200 with TM2 E243 of the neighboring subunit may alter the TM2 tilting angles and induce a conformational change.

### Why only the β1–β2 loop and pre-TM1

Four regions from the EC domain (β1–β2, β8–β9, β10, and the Cys-loop) and two regions from the TM domain (pre-TM1 and the TM2-TM3 linker) comprise the coupling interface between the EC and TM domains of GLIC and other pLGICs. Previous studies on Cys-loop receptors have shown that these regions, either individually or in combination, mediate the transduction of agonist binding to channel gating [4]–[6], [26], [28], [29], [34], [35].

In the context of the PMT model, paths to TM2 through either the β1–β2 loop or pre-TM1 have higher probabilities than paths through other loops, such as the Cys-loop and the TM2-TM3 linker. While these loops were not detected in the highest probability paths, this does not imply that such loops are not important. The PMT model has a limitation in that it only considers the number of atom-atom contacts for the probability of passing a signal from one residue to another. Consequently, Van der Waals interactions are weighted more heavily than Coulombic interactions. For Cys-loop receptors, the importance of salt bridges at the interface of the EC and TM domains has been well documented [6], [34], [36]. Thus, our results should not be interpreted to rule out the functional contribution of the Cys-loop and the TM2-TM3 linker. Rather, these results explicitly demonstrate the importance of the β1–β2 loop and pre-TM1 in the signaling pathways.

### The signaling pathway within the muscle-type nAChR

**Figure 4:**
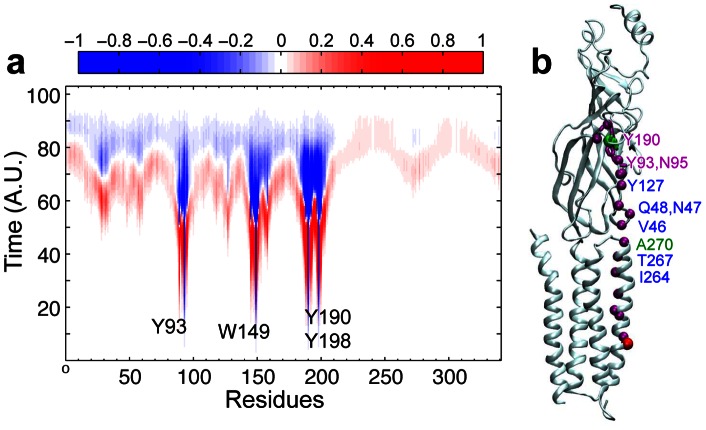
Trajectory of the probability flux and the highest probability path in nAChR (PDB code: 2BG9). (**a**) Trajectory of the probability flux over time for each residue of the α1 nAChR upon perturbation to the agonist-binding site (Y93, W149, Y190, and Y198). The color denotes the normalized intensity of the flux. Positive and negative signs describe the net signal flow into and out of the residue, respectively. (**b**) The signaling path with highest probability between Y190 of the C loop and the pore-lining residue L251 (9′) in the α1 nAChR. Perturbation starting and ending points are shown in green and red spheres, respectively. Residues comprising the path are shown in purple spheres. The labeled residues were identified previously in the mutagenesis and functional studies for transferring energy from the extracellular domain to the channel gating [25], [27], .

The results from the PMT model depend heavily on the protein structure. Therefore, what we observed on GLIC is expected to be applicable to the homologous Cys-loop receptors. To confirm this is the case, we performed the same calculations on the muscle-type nAChR (PDB code: 2BG9). The advantage of using the muscle-type nAChR is not only the availability of the structure, but also the availability of extensive experimental data [6], [25]–[28], [37]–[39]. The initial perturbation was placed at the agonist binding site, namely residues α1-Y93, α1-W149, α1-Y190, and α1-Y198 (Fig. 4). The results from Yen's algorithm show that these residues in the binding site are well coupled, as they pass signal to each other along the highest probability paths. Thus we examined the representative pathway between Y190 and L251 (L9′).

Despite the inclusion of adjacent subunits in the calculations, the initial perturbation signal traveled only through an intra-subunit path via the β1–β2 loop to reach the channel gate. More interestingly, when the path was constrained between Y190 and the channel gate of either adjacent subunit, the signal still traveled to TM2 within the same subunit before ending at the channel gate residue of the adjacent subunit. This is presumably due to tighter TM2 helical packing in the closed-channel nAChR structure versus the open-channel GLIC structure. The intra-subunit path for the nAChR is similar to the intra-subunit path observed for GLIC (Figs. 3a and 3d). Furthermore, residues along the pathway were previously suggested for signal propagation in experimental studies (Fig. 4b) [25], [27], [37]–[40]. The observed pathway is well supported by experimental data.

### Concluding remarks

Using the PMT model in combination with Yen's algorithm, we revealed multiple pathways for signal transduction from the EC domain to the channel gate. While the EC-TM interfacial structural elements (such as the Cys-loop, the β1–β2 loop, pre-TM1, and the TM2-TM3 linker) are expected to play roles in the signal transduction, we only found the β1–β2 loop or pre-TM1 in the signal transduction pathways of the highest probability upon different perturbations to the EC domain. Paths involving the β1–β2 loop can be either within a subunit or between adjacent subunits, but paths involving pre-TM1 are exclusively between adjacent subunits. In the past, signaling involving pre-TM1 has been attributed to pre-TM1 coupling with other loops at the EC-TM interface. Our data suggest that pre-TM1 can directly couple with TM2 of the adjacent subunit, providing a new insight into the allosteric signaling mechanisms of pLGICs.

## Materials and Methods

### PMT calculations

PMT calculations were performed on the pentameric GLIC using the online server (http://gila-fw.bioengr.uic.edu/lab/tools/pmtmodel/). Details of the PMT model were provided in the previous publication [21]. Briefly, the Markovian transition model [16] was used to investigate how a given perturbation is transmitted through a protein network over time. At each time step, the perturbation is transmitted from residue *i* to residue *j* with a probability m*_ij_*, an element in the Markovian transition matrix **M** =  {m*_ij_*}*_N×N_*, where *N* is the total number of residues in the protein and 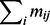. Each residue is represented as a single node in the model. The m*_ij_* values are computed from the atomistic (no hydrogens) structure according to m*_ij_* =  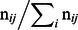, where n*_ij_* is the number of atom-atom contacts between residues *i* and *j*. Two atoms from different residues are considered in contact if the Euclidean distance between the two atoms is less than or equal to 4.5 Å, the cutoff that consistently displayed the fastest signal propagation for all tested perturbation sites [16], [21]. The initial perturbation, ***p***(0), is defined by a set of probabilities {*p_i_*(0)}*_N_*, where *p_i_*(0) is the probability mass located at node *i* at time *t* = 0. The signal distribution at time *t* is defined by a vector ***p***(*t*)  =  [*p_1_*(*t*), …, *p_N_*(*t*)]. The probability flow, which depends on both **M** and ***p***(0), provides clues to the signal transduction within the protein under a particular stimulus. The final distribution at equilibrium, ***p***(∞), depends only on **M**, not on ***p***(0). The maximum probability time is defined as the model time required for a residue to reach its maximal probability flux. The master equation describing time-dependent transmission of perturbation is
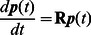
(1)where **R** = **M**–**I**, and **I** is the identity matrix. The Krylov subspace method [41] was used for computing each ***p***(*t*).

The top elementary (or fundamental) signal paths of the highest probability were further elucidated using Yen's algorithm [24] implemented in MATLAB^®^ (http://www.mathworks.com/matlabcentral/fileexchange/32513-k-shortest-path-yens-algorithm). Briefly, for Yen's algorithm, we transformed the Markov transition matrix, **M**, in the PMT model to a “cost” matrix by computing the element-wise inverse of **M**. Yen's algorithm computes the summed cost for transitions between node i and node j. The cost of each transition corresponds to element i,j in the cost matrix. The sequence of nodes that minimizes the cost between the specified starting and ending nodes was determined. The lower the cost is, the higher the probability of the signal path will be.

All the data were processed using MATLAB7.10 (The MathWorkds Inc.). VMD was used to render protein images [42].

The initial perturbation sites were chosen based on our crystal structures and functional measurements of GLIC reported previously (pdb code: 4F8H) [13] and reported below.

### Free energy calculations for the subunit interface

To compare the stability of the subunit interface before and after the NQN mutation in GLIC, we calculated free energy changes for the subunit interface in the crystal structures of the wild type GLIC and the NQN mutant GLIC using the PISA online server (http://www.ebi.ac.uk/msd-srv/prot_int/pistart.html) [43].

### Protein preparation, crystallization, and structure determination of the NQN mutant

The NQN (D91N, E177Q, and D178N) mutation to remove potential salt bridges between the C loop and the complementary side of the adjacent subunit was achieved using site-directed mutagenesis on GLIC with the QuikChange Lightning Kit (Stratagene, Santa Clara, CA) and confirmed by DNA sequencing. The GLIC mutant was expressed in Rosetta(DE3)pLysS (Novagen) and purified as reported in details previously [10], [13], [23]. The pentameric GLIC-NQN mutant in 0.01% (w/v) *n*-tetradecyl-β-D-maltoside from a final purification using size exclusion chromatography was concentrated to ∼10 mg/ml and used for crystallization.

The crystallization and cryo-protection conditions used for the GLIC-NQN mutant were the same as those used previously for GLIC and the GLIC-ketamine complex [13]. The X-ray diffraction data were acquired on beamline 12–2 at the Stanford Synchrotron Radiation Lightsource and processed using the XDS program [44]. The initial structure was solved by molecular replacement using the GLIC-ketamine structure (PDB code: 4F8H) as the starting model. The NQN mutations were made manually on the model with COOT [45]. Phenix (version: 1.8.1) [46] was used for structure refinement. Six detergent and ten lipid molecules were built into well-defined extra electron densities after initial refinement runs. Oxalate molecules degraded from PEG reagents, acetate ions from the crystallization solution, and water molecules were built into the electron densities at the final stages of the refinement with COOT [45]. Non-crystallographic symmetry (NCS) restraints were applied for five subunits in each asymmetric unit. The stereochemical quality of the model was checked with PROCHECK [47] and MolProbity [48]. Crystal structure analysis was performed using Phenix and CCP4 [49]. PyMOL [50] and VMD [42] programs were used for structural analysis and figure preparation.

### Functional measurements of the NQN Mutant

For functional measurements of the NQN mutant, the site-directed mutagenesis was introduced to GLIC in the pTLN vector for expression in *Xenopus laevis* oocytes and confirmed by DNA sequencing. The plasmid DNA was linearized with MluI enzyme (New England BioLabs, Ipswich, MA). Capped complementary RNA was transcribed with the mMESSAGE mMACHINE SP6 kit (Ambion, Austin, TX) and purified with the RNeasy kit (Qiagen, Valencia, CA). The defolliculated stage V-VI oocytes were injected with cRNA (10–25 ng/each) and maintained at 18°C in Modified Barth's Solution (MBS) containing 88 mM NaCl, 1 mM KCl, 2.4 mM NaHCO_3_, 15 mM HEPES, 0.3 mM Ca(NO_3_)_2_, 0.41 mM CaCl_2_, 0.82 mM MgSO_4_, 10 μg/ml sodium penicillin, 10 μg/ml streptomycin sulphate, 100 μg/ml gentamycin sulphate, pH 6.7. Two-electrode voltage clamp experiments were performed on oocytes expressing the NQN mutant at room temperature 16–40 hours after the injection, using a model OC-725C amplifier (Warner Instruments) and a 20-μl recording chamber (Automate Scientific). Oocytes were perfused with ND96 buffer (96 mM NaCl, 2 mM KCl, 1.8 mM CaCl_2_, 1 mM MgCl_2_, 5 mM HEPES, pH 7.4) and clamped to a holding potential of –40 or –60 mV. The ND96 buffer at the lower pH was prepared with the addition of 5 mM MES and HCl. Data were collected and processed using Clampex 10 (Molecular Devices). The data were fit by least squares regression to the Hill Equation using Prism software (Graphpad). The same software was also used for statistic analysis using extra sum-of-squares F-test.

## Supporting Information

Table S1 **Highest probability paths for each residue in the NQN mutation site (D91, E177, D178) and ketamine binding site (N152, D153, D154, F174, L176, K183).**
(PDF)

Table S2 **Number of paths identified for each perturbation in three different ending scenarios.** For subunit B to subunit B, each perturbation has a path that includes the β1–β2 loop, but only 4 of the 9 perturbations produce a path that involves pre-TM1. For subunit B to subunit A, all paths exclusively involve the β1–β2 loop. For subunit B to subunit C, paths involving the β1–β2 loop and pre-TM1 are observed with a slight preference for pre-TM1.
(PDF)
