# Supplementary material for: Signal Transduction Pathways in the Pentameric Ligand-Gated Ion Channels
Source: PLoS One. 2013 May 8;8(5):e64326. doi: 10.1371/journal.pone.0064326 (PMC3648548; doi:10.1371/journal.pone.0064326)
Supplement: Table S1 — Highest probability paths for each residue in the NQN mutation site (D91, E177, D178) and ketamine binding site (N152, D153, D154, F174, L176, K183). (PDF) [file pone.0064326.s001.pdf]

Table S1: Highest probability paths for each residue in the NQN mutation site (D91, E177, D178) and ketamine binding site (N152, D153, D154, F174, L176, K183)

	β1-β2 Loop	Pre-TM1
Subunit B to subunit B		
D91	V90, V89, D88, A87, D86, S107, A108, R109, **T36, A34, K33**, T244, E243, N239, I236, I233	N/A
D178	E177, L176, Y23, N152, V155, F156, P113, L114, **E35, A34, K33**, T244, E243, N239, I236, I233	E177, L176, Y23, N152, V155, F156, T158, G159, **Q193, Y194, F195, S196, N**200, E243, N239, I236, I233
E177	L176, Y23, N152, V155, F156, P113, L114, **E35, A34, K33**, T244, E243, N239, I236, I233	L176, Y23, N152, V155, F156, T158, G159, **Q193, Y194, F195, S196, N**200, E243, N239, I236, I233
N152	V155, F156, L157, **L30, D31, K33**, T244, E243, N239, I236, I233	N/A
D153	D154, F156, L157, **L30, D31, K33**, T244, E243, N239, I236, I233	N/A
D154	F156, L157, **L30, D31, K33**, T244, E243, N239, I236, I233	N/A
F174	N173, A172, K183, Y129, H127, L126, **F37, T36, A34, K33**, T244, E243, N239, I236, I233	A175, L176, Y23, N152, V155, F156, T158, G159, **Q193, Y194, F195, S196, N**200, E243, N239, I236, I233
L176	Y23, N152, V155, F156, P113, L114, **E35, A34, K33**, T244, E243, N239, I236, I233	Y23, N152, V155, F156, T158, G159, **Q193, Y194, F195, S196, N**200, E243, N239, I236, I233
K183	Y129, H127, L126, **F37, T36, A34, K33**, T244, E243, N239, I236, I233	N/A
Subunit B to subunit A		
D91	V90, V89, D88, A87, D86, R85, A84, N83, E82, Y28, S29, **L30**, **D31**, **K33**, T244, E243, N239, I236, I233	N/A
D178	E177, L176, Y23, L24, I25, E26, C27, Y28, S29, **L30**, **D31, K33**, T244, E243, N239, I236, I233	N/A
E177	L176, Y23, L24, I25, E26, C27, Y28, S29, **L30**, **D31, K33**, T244, E243, N239, I236, I233	N/A
N152	V155, F156, L157, L**30, D31, K33**, P247, K248, N245, T244, E243, N239, I236, I233	N/A
D153	D154, F156, L157, L**30, D31, K33**, P247, K248, N245, T244, E243, N239, I236, I233	N/A
D154	F156, L157, L**30, D31, K33**, P247, K248, N245, T244, E243, N239, I236, I233	N/A
F174	A175, L176, Y23, L24, I25, E26, C27, Y28, S29, **L30**, **D31, K33**, T244, E243, N239, I236, I233	N/A
L176	Y23, L24, I25, E26, C27, Y28, S29,, **L30**, **D31, K33**, T244, E243, N239, I236, I233	N/A
K183	Y129, N80, V81, E82, Y28, S29, **L30**, **D31**, **K33**, T244, E243, N239, I236, I233	N/A
Subunit B to subunit C		
D91	V90, V89, D88, R105, V79, N80, V110, F37, **T36, E35, A34, K33**, T244, E243,	N/A

	N239, I236, I233	
D178	E177, L176, Y23, N152, V155, F156, P113, L114, **E35, A34, K33**, T244, E243, N239, F238, I236, I233	R179, R133, V132, I131, L130 Y129, H127, T125, Q124, S123, D122, F121, **Y194, F195, S196,** N200, E243, N239, I236, I233
E177	L176, Y23, N152, V155, F156, P113, L114, **E35, A34, K33**, T244, E243, N239, F238, I236, I233	L176, Y23, N152, V155, F156, T158, G159, **Q193, Y194, F195, S196**, N200, E243, N239, F238, I236, I233
N152	V155, F156, T158, P113, L114, **E35, A34, K33,** T244, E243, N239, I236, I233	V155, F156, T158, G159, **Q193, Y194, F195, S196**, N200, E243, N239, I236, I233
D153	D154, F156, T158, P113, L114, **E35, A34, K33,** T244, E243, N239, I236, I233	D154, F156, T158, G159, **Q193, Y194, F195, S196**, N200, E243, N239, I236, I233
D154	F156, T158, P113, L114, **E35, A34, K33,** T244, E243, N239, I236, I233	F156, T158, G159, **Q193, Y194, F195, S196**, N200, E243, N239, I236, I233
F174	N/A	N173, A172, P171, K170, V168, A167, T166, F165, S164, E163, I162, D161, **Q193, Y194, F195, S196**, N200, E243, N239, I236, I233
L176	Y23, N152, V155, F156, P113, L114, **E35, A34, K33**, T244, E243, N239, F238, I236, I233	Y23, N152, V155, F156, T158, G159, Q**193, Y194, F195, S196**, N200, E243, N239, F238, I236, I233
K183	N/A	Y129, H127, T125, Q124, S123, D122, F121, **Y194, F195, S196**, N200, E243, N239, I236, I233

*Residues colored black, red, and green belong to subunits B, A, and C, respectively.

*Residues in the β1-β2 loop or pre-TM1 are highlighted in bold
