# Supplementary material for: Signal Transduction Pathways in the Pentameric Ligand-Gated Ion Channels
Source: PLoS One. 2013 May 8;8(5):e64326. doi: 10.1371/journal.pone.0064326 (PMC3648548; doi:10.1371/journal.pone.0064326)
Supplement: Table S2 — Number of paths identified for each perturbation in three different ending scenarios. For subunit B to subunit B, each perturbation has a path that includes the β1–β2 loop, but only 4 of the 9 perturbations produce a path that involves pre-TM1. For subunit B to subunit A, all paths exclusively involve the β1–β2 loop. For subunit B to subunit C, paths involving the β1–β2 loop and pre-TM1 are observed with a slight preference for pre-TM1. (PDF) [file pone.0064326.s002.pdf]

Table S2. **Number of paths identified for each perturbation in three different ending scenarios.** For subunit B to subunit B, each perturbation has a path that includes the β1-β2 loop, but only 4 of the 9 perturbations produce a path that involves pre-TM1. For subunit B to subunit A, all paths exclusively involve the β1-β2 loop. For subunit B to subunit C, paths involving the β1-β2 loop and pre-TM1 are observed with a slight preference for pre-TM1.

	**β1-β2 Loop**	**Pre-TM1**
**Subunit B to subunit B**		
D91	10	0
E177	2	8
D178	2	8
N152	10	0
D153	10	0
D154	10	0
F174	8	2
L176	2	8
K183	10	0
	71%	29%
**Subunit B to subunit A**		
D91	10	0
E177	10	0
D178	10	0
N152	10	0
D153	10	0
D154	10	0
F174	10	0
L176	10	0
K183	10	0
	100%	0%
**Subunit B to subunit C**		
D91	10	0
E177	2	8
D178	1	9
N152	3	7
D153	3	7
D154	3	7
F174	0	10
L176	2	8
K183	0	10
	27%	73%
**Overall**	**66%**	**34%**
